# An unusual presentation of a urethral diverticulum as a vaginal wall mass: a case report

**DOI:** 10.1186/1752-1947-7-171

**Published:** 2013-07-01

**Authors:** Megan Billow, Rebecca James, Kimberly Resnick, Adonis Hijaz

**Affiliations:** 1 Department of OB/GYN, University Hospitals Case Medical Center, Cleveland, OH 44106, USA; 2 Department of OB/GYN, Division of Gynecologic Oncology, University Hospitals Case Medical Center, Cleveland, OH 44106, USA; 3 Department of Urology, University Hospitals Case Medical Center, Cleveland, OH 44106, USA

**Keywords:** Urethral Diverticulum, Vaginal Cancer, Vaginal Mass

## Abstract

**Introduction:**

The diagnosis of urethral diverticulum can be challenging given the vague or absent presenting symptoms. In addition, vaginal cancer can present with elusive symptoms--some parallel to urethral diverticula. A case of a bleeding ulcerated mass anticipated to be a vaginal cancer was instead identified as a benign urethral diverticulum. To the best of our knowledge, this is the first case report of a benign urethral diverticulum presenting as a bleeding, necrotic ulcerated mass.

**Case presentation:**

A 52 year-old multiparous African-American woman presented with a 2-day history of heavy vaginal bleeding passing large clots and suprapubic pain. A pelvic examination revealed blood clots in the vagina along with a friable, fibrous ulcerated lesion on the anterior suburethral vagina, just left of the midline measuring 4 × 2cm. Initially, this mass was considered to be a vaginal cancer. Intraoperative diagnosis of a benign urethral diverticulum was made.

**Conclusions:**

The diagnosis of urethral diverticula based on the vast array of presenting symptoms, is difficult. This original case report may benefit both gynecologic oncologists and female pelvic surgeons and reconstructive surgeons to keep urethral diverticulum in the differential diagnosis when faced with a bleeding midline anterior vaginal mass. This unusual presentation of a urethral diverticulum demonstrates how similarly it may present to a vaginal cancerous mass.

## Introduction

A urethral diverticulum is a permanent, epithelialized, non-muscular, sac-like cavity projecting into the periurethral fascia arising from the posterior urethral lumen with an incidence of 1 to 6% in the general population [1]. They range from 3mm to 3cm in diameter. The most widely accepted hypotheses attribute urethral diverticulum formation to repeated infection of the periurethral glands with subsequent obstruction and abscess formation, which then form epithelialized and urothelium-lined outpouchings. Other proposed etiologies include childbirth trauma, iatrogenic causes, and urethral instrumentation [2].

They present at any age, but more commonly in the second and third decade of life with nonspecific symptoms. Due to vague or absent symptoms, patients are often misdiagnosed. Frequency, urgency, dysuria, dyspareunia, and post-void dribbling are commonly pathognomonic of urethral diverticulum [3]. Palpating the anterior vaginal wall may reveal a painful mass because urethral diverticula usually present ventrally.

Similarly, vulvar cancer presents with nonspecific symptoms and is often difficult to diagnose. The most common presenting symptoms include a visible or palpable mass, bleeding, ulceration, vaginal discharge, pain, dysuria, and pruritus. When vulvar cancers are diagnosed in an advanced stage, it is often attributed to the vague presentation and delay in diagnosis. If a vulvar lesion is identified by physical examination, a biopsy is imperative to evaluate for carcinoma [4].

Furthermore, diagnosis can be difficult if a malignant transformation presents within the diverticulum such as several described cases including adenocarcinoma, transitional cell carcinoma, and squamous cell carcinoma [5].

We present the case of a patient who presented with a necrotic, ulcerated, bleeding vaginal mass initially thought to be a vulvar cancer based on the presentation, but found to be a urethral diverticulum.

## Case presentation

A 52-year-old multiparous African American woman presented from an outside hospital after syncope. The patient had a 2-day history of heavy vaginal bleeding passing large clots and suprapubic pain. She denied any vaginal pain, dysuria, hematuria, or frequency. She had a remote history of a total abdominal hysterectomy and bilateral salpingo-oophorectomy secondary to leiomyomata.

**Figure 1:**
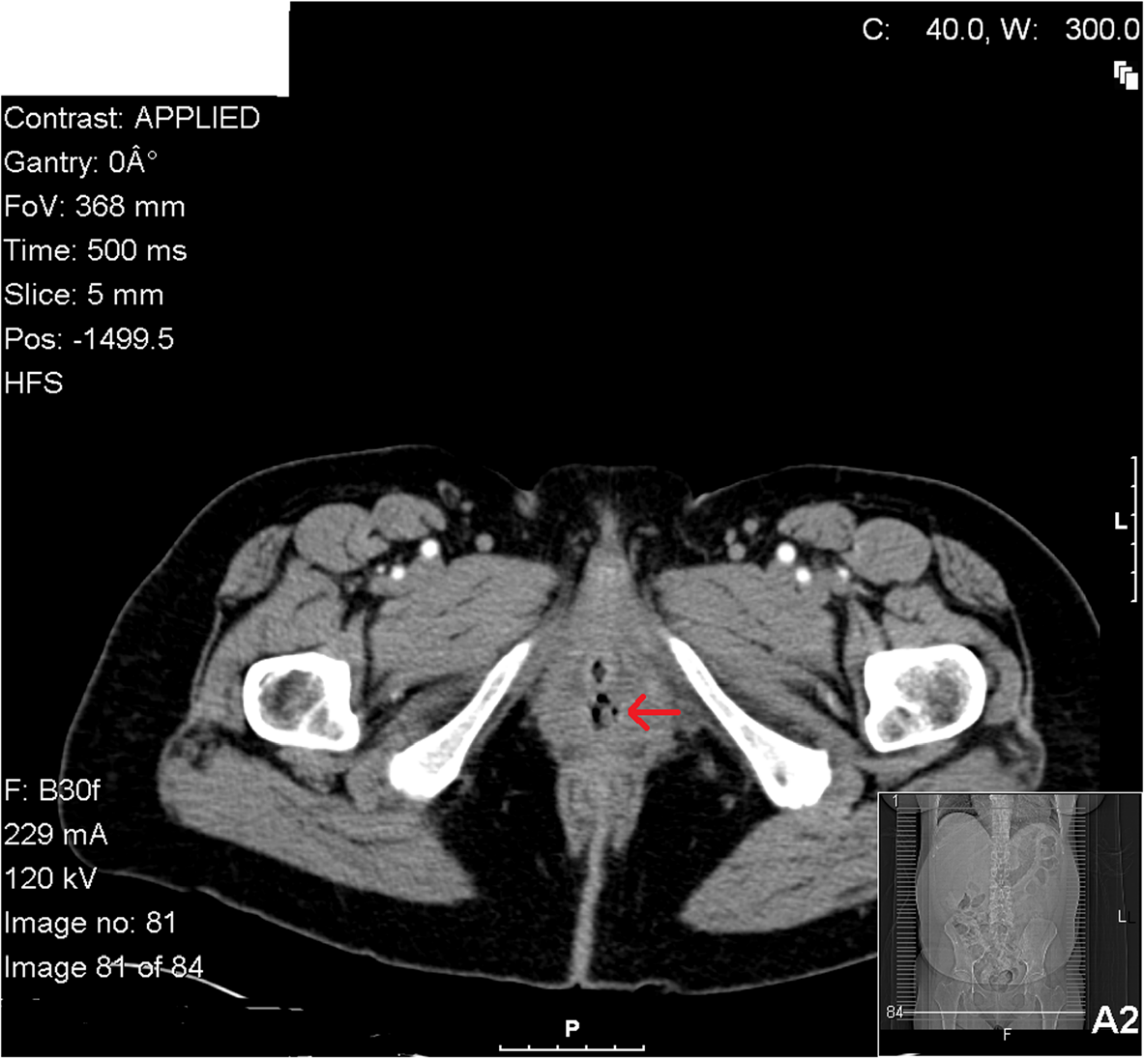
**Computed tomography scan of urethral diverticulum.** Red arrow indicates urethral diverticulum.

The initial pelvic examination revealed blood clots in the vagina along with a friable, fibrous ulcerated lesion on the anterior suburethral vagina, just left of the midline measuring 4 × 2cm. The lesion was actively bleeding and necrotic, but did not appear to extend beyond the sidewall. Biopsies of the specimen were insufficient for diagnosis revealing blood and necrotic material. A computed tomography scan revealed a soft tissue mass within the lumen of the vagina and an enlarged lymph node located within the right ovarian chain (Figure 1). A staging positron emission tomography scan was performed revealing hypermetabolic activity in the mass and of nodes in the bilateral distal common, left external and bilateral internal iliac chains.

On hospital day (HD) 2, the patient underwent an examination under anesthesia with repeat biopsies. With improved visualization, the ulcerated mass was noted to be 4.5 × 2.5cm in the mucosa of the anterolateral vagina left of the midline parallel to the urethra. Beyond the lesion, inspection of the surrounding vaginal anatomy revealed otherwise normal vaginal mucosa. The parametria and cul-de-sac were normal and smooth bilaterally. Based on this examination and clinical presentation, concern was for vaginal squamous cell carcinoma. However, the pathology was again non-diagnostic.

During HDs 3 through 5, the patient’s bleeding had decreased significantly, and she was asymptomatic. On HD 6, a second examination under anesthesia again revealed biopsies negative for malignancy. A Tonsil clamp was placed into the urethra and visualized through a breach in the vaginal wall around the vaginal mass. The urology service was intraoperatively consulted, and the diagnosis of urethral diverticulum at the 5 o’clock position was confirmed. Cystoscopy confirmed a urethral defect toward the right distal aspect and another area of communication near the 7 o’clock position. The diverticulum was then excised, and a multilayered repair over the Foley catheter was performed.

On postoperative day 9 the patient re-presented with bleeding. An examination under anesthesia revealed several rejected sutures in the necrotic tissue. These were removed, and interrupted mattress sutures were placed to reapproximate the tissue. Final pathology revealed benign squamous mucosa.

In the urology clinic 2 weeks later, a voiding cystourethrogram was performed revealing no extravasation from the urethra or bladder. A physical examination revealed proper vaginal healing and no defects.

## Discussion

Female urethral diverticula often become diagnostic dilemmas. Acquired or congenital, they usually present in women of reproductive age, unlike this post-menopausal patient. Most diverticula result from repetitive or chronic infections of the periurethral glands, which are unresponsive to multiple courses of oral antibiotics, leading to chronic inflammation. This chronic inflammation explains why some cases (such as this presentation) may demonstrate bleeding at the lesion, even in the absence of a urethral stone or malignancy. Huffman [4] likened urethral anatomy to a tree with numerous branches representing the periurethral glands and ducts. A suburethral infection may obstruct these ducts and glands, leading to enlarged retention cysts. These cysts may rupture into the urethral lumen and produce a suburethral diverticulum [3].

The diagnosis of diverticula based on the vast array of presenting symptoms is difficult. Classically, the milking of purulent discharge from the urethra after compressing the suburethral area during examination is highly specific, although poorly sensitive. A urine analysis and culture should be sent; women often complain of recurrent urinary tract infections [1]. Cystoscopy can be performed to thoroughly evaluate the urethral anatomy. Other imaging modalities available for evaluation include double-balloon positive-pressure urethrography, voiding cystourethrography, intravenous urography, ultrasonography, and magnetic resonance imaging.

Presenting symptoms vary including dysuria, dyspareunia, or stress urinary incontinence. Romanzi *et al*. [3] described pain as the primary symptom in 48% of women while another 52% of patients presented with a tender cystic swelling in the anterior vaginal wall. Firm masses found on palpation suburethrally may also heighten the suspicion for calculi or carcinomas [1].

Several case reports have presented malignant diverticula [5]. The presenting symptoms were commonly hematuria and urinary symptoms. To the best of our knowledge, this is the first case report of a benign urethral diverticulum presenting as a bleeding, necrotic ulcerated mass.

## Conclusions

This unusual presentation of a urethral diverticulum emphasizes the importance of keeping a broad differential diagnosis when faced with an anterior midline vaginal mass with symptoms such as bleeding and necrosis. Gynecologic oncologists, female pelvic surgeons, and general gynecologists can all benefit from an understanding of this rare diagnosis and the possibility of vague symptoms upon presentation. Furthermore, this original study highlights that urethral diverticula may mimic other disorders and that symptoms refractory to therapy should prompt consideration of this diagnosis.

## Consent

Written informed consent was obtained from the patient for publication of this manuscript and accompanying images. A copy of the written consent is available for review by the Editor-in-Chief of this journal.

## Competing interest

The authors have no conflicts of interest or financial disclosures.

## Authors’ contributions

MB performed background research, data collection, and manuscript writing and was one of the surgeons during the first operating room case. RJ performed background research, data collection, manuscript writing, editing, and submission, and was a surgeon at the time of diagnosis. AH was the surgeon who diagnosed the urethral diverticulum. KR was the surgeon who initially took the patient to the operating room and performed biopsies. All authors read and reviewed the final manuscript.
